# Biobanks and cancer genome projects in China

**DOI:** 10.1186/1479-5876-10-S2-A51

**Published:** 2012-10-17

**Authors:** Youyong Lv

**Affiliations:** 1 Laboratory of Molecular Oncology, Beijing Cancer Hospital/ Institute, Peking University, Beijing, China

## 

Cancer is the first cause of death in China. Approximately 1.6 million people died of cancer and more than 2.2 millions of new cases were diagnosed each year. In order to reveal the puzzle of genomic alterations and biology of human cancers, the new-generation sequencing technology has been set up and started to carry out large-scale cancer genome study in China. The Chinese Cancer Genome Consortium (CCGC) was organized in August 2008 to launch and coordinate a number of research projects as a publicly-funded network with over 30 university hospitals and research institutions to share a common goal and platform.

The CCGC has done a serial of activities including organization of clinical research teams and working groups, the selection of cancer types and define of research strategies, the technical and bioethical issues for bio-specimen collecting and quality control, which shall be collected using a Standard Operating Procedure (SOP) provided by the CCGC Project Secretary Office following International Cancer Genome Consortium (ICGC). We have proposed the missions and working plan for coming 5 years.

The CCGC has announced approximately 15 types of common cancers in China to be initiated, including gastric, hepatocellular, esophageal, nasopharyngeal, colorectal, bladder, lung, thyroid, breast, renal, ovary, pancreatic cancer, leukemia and glioblastoma. Furthermore, we will focus to optimize the biospecimen collection network and running system for pathological and molecular quality control to support CCGC projects to be healthy growth.

